# Mechanical Loading Induces NRF2 Nuclear Translocation to Epigenetically Remodel Oxidative Stress Defense in Osteocytes

**DOI:** 10.3390/antiox14030346

**Published:** 2025-03-15

**Authors:** Yue Guo, Jing Zhang, Luyu Gong, Na Liu, Qiaoqiao Liu, Zhaojun Liu, Baosheng Guo, Jingping Yang

**Affiliations:** Jiangsu Key Laboratory of Molecular Medicine, Medical School, Nanjing University, Nanjing 210093, China; mg21350031@smail.nju.edu.cn (Y.G.); dg20350040@smail.nju.edu.cn (J.Z.); 502022350012@smail.nju.edu.cn (L.G.); dg21350042@smail.nju.edu.cn (N.L.); dz21350004@smail.nju.edu.cn (Q.L.); 502023350023@smail.nju.edu.cn (Z.L.)

**Keywords:** osteocytes, mechanical loading, NRF2, epigenetic remodeling, pharmacological modulation, oxidative homeostasis

## Abstract

The mechano-responsiveness of osteocytes is critical for maintaining bone health and associated with a reduced oxidative stress defense, yet the precise molecular mechanisms remain incompletely understood. Here, we address the gap by investigating the epigenetic reprogramming that drives osteocyte responses to mechanical loading. We found overall remodeling of antioxidant response under mechanical loading and identified NRF2, a key transcription factor in oxidative stress response, which plays a vital role in the epigenetic remodeling of osteocytes. The results showed that mechanical loading enhanced NRF2 protein stability, promoted its nuclear translocation, and activated osteocyte-specific transcriptional programs. In contrast, pharmacological stabilization of NRF2 failed to fully replicate these effects, underscoring the unique role of mechanical stimuli in modulating NRF2 activity and antioxidant function. Our findings highlight the potential therapeutic limitations of NRF2-stabilizing drugs and suggest that combining pharmacological approaches with mechanical interventions could offer more effective treatments to maintain oxidative homeostasis.

## 1. Introduction

The mechano-responsiveness of osteocytes, the primary bone cells involved in sensing and responding to mechanical loading, is essential for maintaining bone health and integrity [1,2,3]. In parallel, oxidative stress has been recognized as a detrimental factor in bone degeneration and osteocyte dysfunction, contributing to diseases like osteoporosis [4]. Meanwhile, oxidative stress is also a key modulator of cell fate decision in osteoarthritis and osteoporosis [5,6]. Studies showed that regulating oxidative stress-related changes can ameliorate intervertebral disc degeneration (IDD) [7]. All these studies indicate that redox homeostasis plays a pivotal role in osteocyte function. However, the precise molecular mechanisms linking remodeling of oxidative stress response and mechanotransduction remain poorly understood.

Transcriptional remodeling and the gene regulatory network (GRN) [8] are crucial in the mechanical response of osteocytes. Current research indicates that mechanical loading significantly influences gene expression in osteocytes, playing a pivotal role in their mechano-responsiveness [9]. For instance, exercise derepresses the transcription of *Nrf2*, a key transcription factor in oxidative stress response to prevent osteoporosis [10]. Mechanical loading also induces the expression of *Pdpn* and *Ptgs2*, which are essential genes for osteocyte function [11,12,13]. Despite these insights, the regulatory mechanisms of transcriptional remodeling in osteocytes are not well understood.

Mechanical loading can remodel transcriptional regulatory programs in various ways. It can modulate transcription factors (TFs) that bind to the genome, thereby reprogramming gene expression [12,14]. For instance, mechanical loading has been shown to activate YAP in epidermal stem cells and fibroblasts. Additionally, studies indicate that mechanical loading is associated with histone modifications [15]. For example, the effects of mechanical loading depend on H3K9me3 in Chinese hamster ovary cells [16], and HDAC5 plays a role in loading-induced suppression of *Sost* in mature osteocytes [17]. However, the genome-wide epigenetic mechanisms in osteocytes remain unclear, warranting further investigation into this complex process.

Here, we address the gaps in our understanding of how mechanical loading influences oxidative homeostasis in osteocytes. Using MLO-Y4 cells, we investigate the transcriptome and histone modification profiles to elucidate the epigenetic factors that drive osteocyte responses to mechanical loading. Our results revealed significant oxidative stress defense remodeling, with NRF2 emerging as a key regulator. Instead of transcriptional regulation of *Nrf2*, we found that mechanical loading stabilized NRF2 protein levels and promoted its nuclear translocation, leading to enhanced genomic binding of NRF2 and activation of the essential genes of the osteocyte. In contrast, pharmacological stabilization of NRF2 failed to fully replicate these effects. These findings advance our understanding of osteocyte mechanotransduction and highlight the potential for developing integrated therapeutic strategies targeting both mechanical and molecular stimuli to improve osteocyte oxidative homeostasis.

## 2. Materials and Methods

### 2.1. Cell Cultures

The MLO-Y4 cell line (Shanghai Zhong Qiao Xin Zhou Biotechnology Co., Ltd., Shanghai, China, ZQ0954RRID:CVCL_M098) was maintained in DMEM medium (Gibco, Thermo Fisher Scientific, Waltham, MA, USA, 12800-017) with 5% FBS (Gibco, Thermo Fisher Scientific, Waltham, MA, USA, 10270-1106) and 5% NBCS (Gibco, Thermo Fisher Scientific, Waltham, MA, USA, 16010-159) at 37 °C in a 5% (*v*/*v*) CO_2_ humidified incubator.

### 2.2. Mechanical Loading

For the loaded group, MLO-Y4 cells were plated onto UniFlex^TM^ Culture Plates coated with type I collagen (Flexcell International Corporation, Flexcell International Corporation, Burlington, NC, USA), which are 6-well plates with a soft silicone rubber membrane at the bottom of each well. When the cells reached 60% confluency, the plates were subjected to the Flexcell FX5000 Tension System and treated with equiaxial dynamic stretching at 0.5 Hz and 20% strain for 12 h.

### 2.3. RNA-Seq

Cells were lysed using TRIzol (Invitrogen, Thermo Fisher Scientific, Waltham, MA, USA). Each sample contained more than 1.5 µg of total RNA. Ribosomal RNA was depleted to enrich the total RNA. The library was constructed and sequenced by Allwegene Company. Two replicates were performed for each group.

### 2.4. ChIP-Seq

ChIP assays were performed as previously described [18]. MLO-Y4 cells were fixed by 1% formaldehyde solution (Sigma, St. Louis, MO, USA, F8775-25ML) and quenched with 1.25M glycine (Amresco, Solon, OH, USA, 200-272-2). Chromatin was collected and sheared using the Bioruptor Plus with 30 s on and 30 s off for 18 cycles. Immunoprecipitation was performed using 3–5 μg of H3K27ac (Abcam, ab4729, RRID:AB_2118291, Cambridge, UK), H3K4me3 (Abcam, ab8580, Cambridge, UK, RRID:AB_306649), or NRF2 (Proteintech, Rosemont, IL, USA, 80593-1-RR, RRID:AB_2918904) antibodies. DNA was purified using the DNA Clean & Concentrator-5 kit (Zymo, Irvine, CA, USA D4014). Libraries were then constructed from ChIP samples using the NEBNext Ultra DNA Library Prep Kit for Illumina (NEB, Ipswich, MA, USA, E7370) and sequenced on a NovaSeq by Annoroad Gene Tech (Beijing) Co., Ltd. (Beijing, China). Two biological replicates were performed for each group.

### 2.5. RNA-Seq Analysis

RNA-seq analysis was performed as previously described [19]. The adaptor-trimmed and quality-filtered reads were mapped to the mm10 genome using HISAT2 (version 2.1.0). Transcript assembly was performed using StringTie (version 1.3.5). The output files from StringTie were used to generate the count matrices for genes by executing prepDE.py. Differential expression was analyzed using DESeq2 (version 1.32.0), with differentially expressed genes identified based on an adjusted *p*-value (padj) of less than 0.01. DESeq2 (version 1.32.0) was also used to perform principal component analysis (PCA) on the unloaded and loaded groups. For visualization of transcription signals obtained from the transcriptome data, deduplicated BAM files were converted into bedgraph files using bedtools (v2.26.0), followed by sorting and conversion to visualizable bigwig files using bedtools (v4). Gene expression levels were then visualized using the Integrative Genomics Viewer (IGV).

### 2.6. ChIP-Seq Analysis

The adaptor-trimmed and quality-filtered reads were aligned to the mm10 using Bowtie2 [20] with default parameters, and uniquely mapped reads were used for peak calling with MACS2 [21]. The NRF2 ChIP-seq data in astrocytes SRR25281578 and SRR25281569, or in human aortic endothelial cell (HAEC) GSM2394418 were processed exactly the same as that in MLO-Y4. A pairwise comparison of NRF2 peaks in MLO-Y4 and astrocytes was performed via peak overlapping by “intersectBed” of “bedtools”. The heat maps of ChIP-seq results were generated by Seqplots (Bioconductor package) [22]. De novo motifs were identified using the “findMotifsGenome.pl” function in Homer with the parameter “–size given” for NRF2-binding regions [23]. The R package ChIPseeker (version 1.28.3) was used for annotating the distribution of enriched ChIP-seq peaks.

### 2.7. Gene Functional Analysis

Metascape online software (https://metascape.org/gp/index.html#, accessed on 12 December 2023) was used to analyze the different functions of the differentially expressed genes obtained from different groups. Gene Set Enrichment Analysis (GSEA) (version 4.3.2) was used to evaluate the enrichment of the NRF2-related gene set including WP2884 and WP4357. GREAT (http://great.stanford.edu/public/html/index.php, accessed on 4 July 2024 and 21 February 2025) was used to analyze the biological function of NRF2 peaks.

### 2.8. Western Blot

The total protein was extracted from cells using highly efficient RIPA lysis buffer (Solarbio, Beijing, China, R0010). For the extraction of nucleus or cytoplasmic proteins, the NE-PER^®^ Nuclear or Cytoplasmic Extraction Kit (Keygen Biotech, Nanjing, Jiangsu, China, KGP150) was used. Proteins were separated using a 10% Color PAGE Gel Rapid Preparation Kit (Epizyme, Shanghai, China) under reducing conditions. The used antibodies were mouse NRF2 antibody (1:5000, Proteintech, Rosemont, IL, USA, 80593-1-RR, RRID:AB_2918904); mouse anti-Lamin B1 antibody (1:1000, Abcam, Ab16048, RRID:AB_443298); mouse beta-Actin (13E5) Rabbit mAb (1:1000, Cell Signaling Technology, 4970, RRID:AB_2223172); and Anti-rabbit IgG, HRP-linked Antibody (1:5000, Cell Signaling Technology, 7074, RRID:AB_2099233). Chemiluminescent signals were detected using the SuperFemto ECL Chemiluminescence Kit (Vazyme, Nanjing, Jiangsu, China, E412-01), quantified using the Tanon Imaging System, and quantified with ImageJ (version 1.54f).

### 2.9. Quantitative Real-Time Polymerase Chain Reaction (qRT-PCR)

Total RNA was isolated using the MiniBEST Universal RNA Extraction Kit (TAKARA, Shiga, Japan, 9767) according to the manufacturer’s instructions. The mRNA was reverse-transcribed using the 5× PrimeScript RT Master Mix (TAKARA, Shiga, Japan, RR036A) from 1 μg of total RNA. qPCR was performed using cDNA with Power SYBR Green PCR Master Mix (Life Technologies LTD, Carlsbad, CA, USA, 4367659). *Actb* was used as an internal control. The primer sequences are listed in Supplementary Table S1.

### 2.10. Statistical Analysis:

Differential expression analysis in panel A of figure in Section 3.3 was conducted using edgeR version 3.26.8, and adjusted *p*-values (FDR) were calculated by using “p.adjust” in R. For panel B–F of figures in Section 3.3, the Shapiro–Wilk test was used to assess normality. The homogeneity of variance was evaluated using the F test for panel B,D–F of figures in Section 3.3 and Levene’s test for panel C of figures in Section 3.3. All data in these figures met the assumptions of normal distribution and homogeneity of variance. For comparison, a two-tailed Student’s *t*-test was used for panel B,D–F of figures in Section 3.3, and one-way ANOVA followed by Bonferroni’s multiple comparison test was used for panel C of figures in Section 3.3. Statistical significance levels were denoted as follows: * *p* < 0.05, ns *p* > 0.05.

## 3. Results

### 3.1. Mechanical Loading Remodels the Transcriptional and Epigenetic Profile of Oxidative Homeostasis in Osteocytes

**Figure 1:**
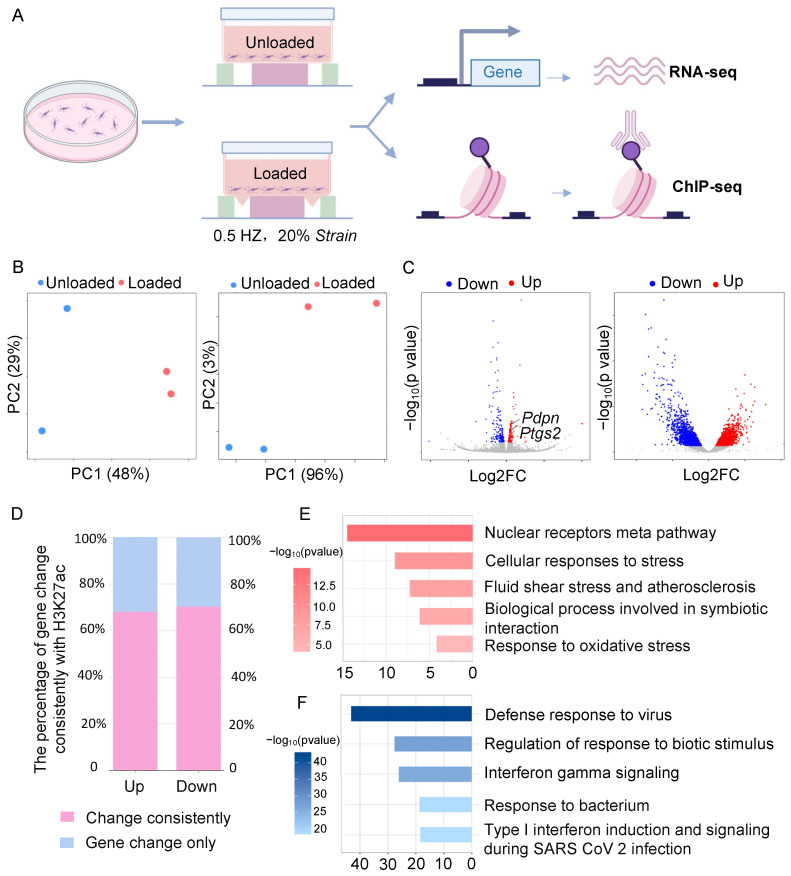
Mechanical loading treatment and multi-omics measurement of osteocytes under mechanical loading. (**A**) Experimental design workflow of mechanical loading of MLO-Y4 cells. (**B**) Principal component analysis (PCA) of RNA-seq profiles (left) and H3K27ac ChIP-seq profiles (right). (**C**) Volcano plot of significantly differentially expressed genes (left, *p* adjusted value < 0.01) and significantly differential H3K27ac peaks (right, *p* adjusted value < 0.01). (**D**) Percentage of differentially expressed genes with consistently changed H3K27ac signals. (**E**) Enrichment of functional pathways for significantly upregulated genes under mechanical loading. (**F**) Enrichment of functional pathways for significantly downregulated genes under mechanical loading.

To investigate the effects of mechanical loading on osteocytes, we used MLO-Y4 cells and subjected them to mechanical loading. We then profiled their transcriptome and histone modification profiles, focusing on H3K27ac (Figure 1A). Both the transcriptome and H3K27ac distribution showed significant remodeling. Principal component analysis (PCA) revealed high reproducibility within groups and significant differences between the unloaded and loaded groups (Figure 1B). Mechanical loading led to the significant upregulation of 169 genes and downregulation of 192 genes, including known targets such as E11 (encoded by *Pdpn*) and *Ptgs2* (Figure 1C). Additionally, the H3K27ac distribution was significantly altered, with thousands of H3K27ac peaks showing changes, indicating extensive epigenetic remodeling (Figure 1C). The integration of transcriptional and epigenetic profiles revealed that most genes (70%) with altered transcription were also associated with changes in epigenetic regulation (Figure 1D). Consistent with previous understanding [24,25], mechanical loading significantly remodeled stress response, especially upregulated antioxidative response (Figure 1E). Meanwhile, it downregulated immune response (Figure 1F). These results confirm that mechanical loading can remodel both the transcriptome and epigenetic regulation of osteocytes, highlighting the intricate interplay between mechanical stimuli and overall stress response.

### 3.2. NRF2 Plays a Vital Role in the Epigenetic Remodeling of Osteocytes

**Figure 2:**
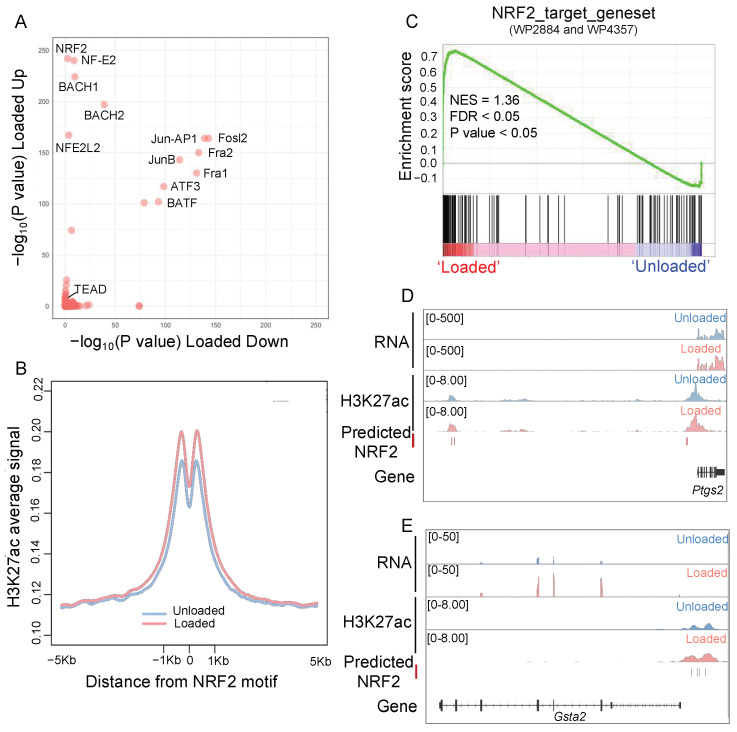
NRF2 is the core transcription factors involved in osteocytes response to mechanical loading. (**A**) Motif enrichment on significantly upregulated and downregulated H3K27ac peaks under mechanical loading. (**B**) The distribution of H3K27ac signals at the predicted NRF2 motif. (**C**) GSEA enrichment shows NRF2 target genes (WP2884 and WP4357) were significantly upregulated under mechanical loading. (**D**) Genome browser view of *Ptgs2* locus with gene expression, H3K27ac signals, and predicted NRF2 motif. The regulatory elements with NRF2 motif are highlighted by the pink bars. (**E**) Genome browser view of the *Gsta2* locus as in (**D**).

To identify key drivers of regulatory remodeling under mechanical loading, we investigated the motif enrichment of H3K27ac remodeling, which reflected transcriptional regulation. Unlike other systems, where the YAP/TAZ-TEAD motif plays a significant role under mechanical loading [12], these motifs were not extensively involved in osteocytes (*p*-value = 1). Instead, the NRF2 motif family was specifically enriched in significantly upregulated H3K27ac peaks, while Fos/AP1 motifs were involved in dynamic peaks in both directions (Figure 2A).

Examining the de novo NRF2 motif, we found that H3K27ac signals were enhanced at predicted NRF2 motifs under mechanical loading (Figure 2B). Additionally, gene set enrichment analysis (GSEA) of NRF2 target gene sets confirmed that NRF2 regulation was significantly activated in osteocytes subjected to mechanical loading (Figure 2C). For instance, *Ptgs2*, the important mechanical target gene in osteocytes, contained predicted NRF2 motifs at its transcriptional regulatory region and exhibited both increased H3K27ac signals at the transcriptional regulatory region and elevated transcription levels (Figure 2D and Figure S1A). The gene *Gsta2*, which is important for oxidative stress protection, showed an increase in H3K27ac signals, as well as upregulated transcription levels under mechanical loading (Figure 2E and Figure S1B). These findings indicate that NRF2 plays a crucial role in the transcriptional regulatory remodeling of osteocytes under mechanical loading, regulating genes essential for both oxidative defense and osteocyte function.

### 3.3. Mechanical Loading Promotes Nucleus Translocation of NRF2 in Osteocytes

**Figure 3:**
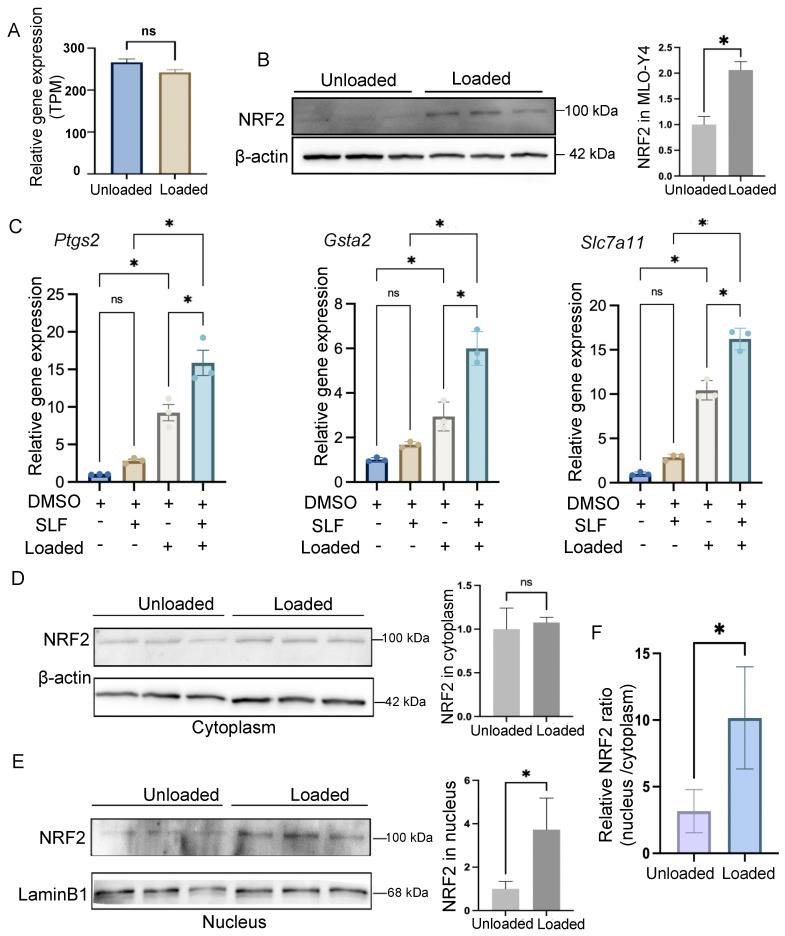
Mechanical loading promotes NRF2 nucleus localization. (**A**) The mRNA levels of *Nfe2l2*. ns *p* > 0.05. (**B**) Western blot analysis of the total protein isolated from cells without or with mechanical loading (left) and the quantification of the protein levels (right, n = 3). * *p* < 0.05. (**C**) mRNA expression of NRF2 target genes *Ptgs2*, *Gsta2*, and *Slc7a11* under treatment of SLF (NRF2 stabilizer) and/or mechanical loading (n = 3, * *p* < 0.05). (**D**,**E**) Analysis of the cytoplasm (**D**) or nucleus (**E**) protein levels. Western blot of protein levels (left) and the quantification of the protein levels (right) from cells without or with mechanical loading (n = 3, ns *p* > 0.05, * *p* < 0.05). (**F**) The ratio of NRF2 protein in the nucleus versus cytoplasm. The level of NRF2 protein is normalized by the level of β-actin in the cytoplasm or the level of Lamin B1 in the nucleus. * *p* < 0.05.

The multi-omics analysis revealed the significant regulatory role of NRF2 in osteocytes under mechanical loading. Previous studies have indicated decreased transcription of NRF2 in bone diseases [10]. However, in osteocytes subjected to mechanical loading, we did not observe any transcriptional alteration of *Nfe2l2*, the gene encoding NRF2 (Figure 3A), suggesting that the regulatory role of NRF2 in osteocytes is independent of its transcriptional change.

To investigate further, we examined the protein levels of NRF2 and found a significant increase in NRF2 protein in osteocytes under mechanical loading (Figure 3B). This prompted us to explore whether this increase in NRF2 protein could mimic the effects of mechanical loading. We treated the cells with sulforaphane (SLF), a known stabilizer of NRF2, to maintain high NRF2 protein levels. We then assessed the expression of NRF2 target genes, including *Ptgs2*, *Gsta2*, and *Slc7a11*. While SLF treatment combined with mechanical loading further enhanced the expression of these genes, SLF alone did not increase their expression (Figure 3C). These findings suggested that simply increasing NRF2 protein levels was not sufficient to replicate the effects of mechanical loading.

Given these observations, we hypothesized that mechanical loading might also promote the nucleus translocation of NRF2, a necessary step for its regulatory function. To test this, we extracted cytoplasmic and nucleus proteins separately and measured the levels of NRF2. While there was no significant difference in cytoplasmic NRF2 levels between loaded and unloaded conditions (Figure 3D), we observed a significant accumulation of NRF2 in the nucleus under mechanical loading (Figure 3E). The ratio of NRF2 protein levels in the nucleus versus the cytoplasm was significantly higher under mechanical loading in osteocytes (Figure 3F).

These results indicated that mechanical loading not only stabilized NRF2 protein levels but also promoted its nucleus translocation, enabling its regulatory functions. In contrast, pharmacologic stabilization of NRF2 alone could not mimic the comprehensive effects of mechanical loading on osteocytes.

### 3.4. NRF2 Exerts Both Shared and Cell Type-Specific Function Through Binding to the Genome of Osteocytes

**Figure 4:**
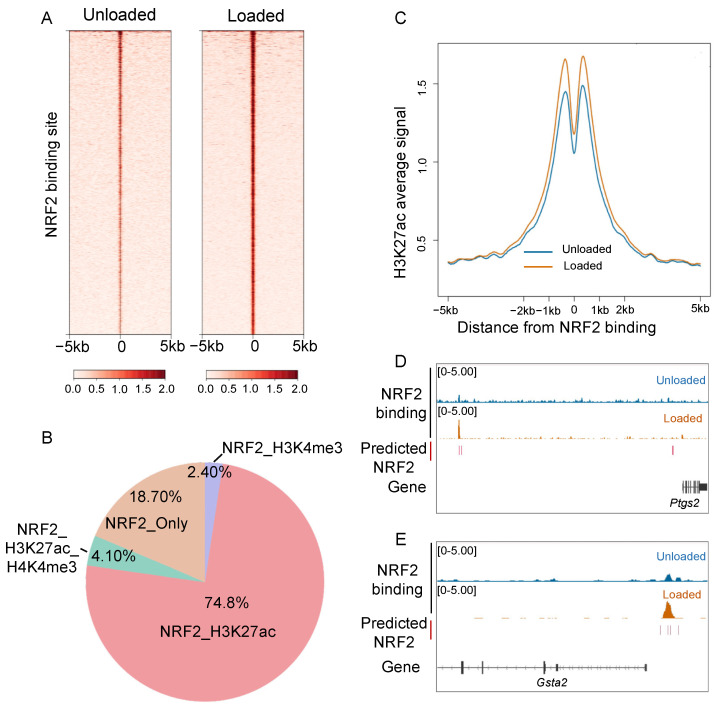
Mechanical loading enhances NRF2 binding to the genome of osteocytes. (**A**) The binding of NRF2 in unloaded and loaded cells. (**B**) The distribution relationship of NRF2 binding sites overlapping with characteristics in the form of H3K4me3 and/or H3K27ac peaks in MLO-Y4 cells. (**C**) The signals of H3K27ac around NRF2 binding sites in cells without or with mechanical loading. (**D**,**E**) Genome browser view of the NRF2 predicted motif, NRF2 binding without mechanical loading, and NRF2 binding with mechanical loading at the *Ptgs2* locus (**D**) and the *Gsta2* locus (**E**). The bars highlight the same regulatory elements as in Figure 2D,E.

To deepen our investigation into the regulatory role of NRF2 in mechanically loaded osteocytes, we performed ChIP-seq analysis of NRF2. The results revealed a significant increase in NRF2 binding to the genome of osteocytes under mechanical loading (Figure 4A). Importantly, 81.3% of NRF2 binding sites were found to contain the histone modifications H3K27ac or H3K4me3, with the majority (74.8%) overlapping with H3K27ac signals (Figure 4B), suggesting these sites acted as potential regulatory elements.

To assess the functional implications of NRF2 binding, we examined the H3K27ac signal around NRF2 binding sites and found a significant increase under mechanical loading (Figure 4C), indicating a correlation between NRF2 binding and H3K27ac enhancement. For instance, at the *Ptgs2* and *Gsta2* locus, ChIP-seq confirmed NRF2 binding at the transcriptional regulatory region, coinciding with enhanced H3K27ac signals and increased expression of *Ptgs2* and *Gsta2* (Figure 4D,E). These findings underscore that mechanical loading-induced nucleus translocation of NRF2 enhances its genomic binding capacity, thereby remodeling transcriptional regulation in osteocytes.

**Figure 5:**
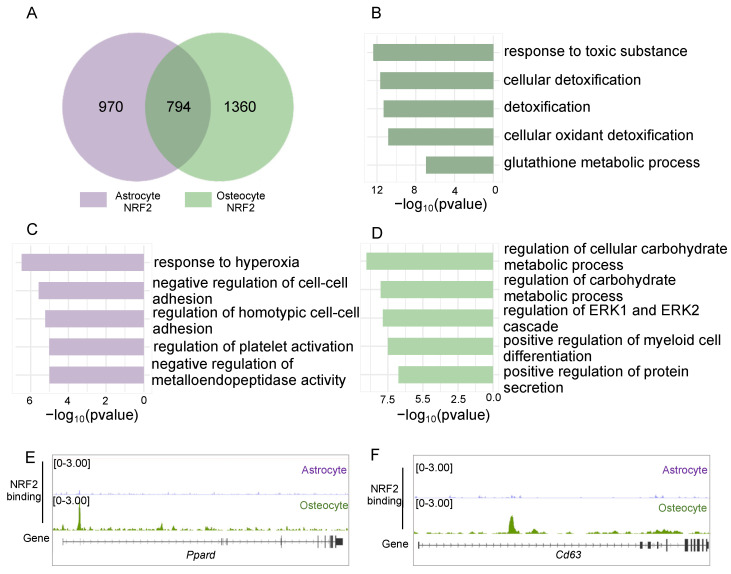
NRF2 exerts cell-type-specific role in osteocytes. (**A**) Comparison of NRF2 binding sites between osteocytes and astrocytes. (**B**–**D**) Gene ontology and pathway enrichment of shared (**B**), astrocyte-specific (**C**), and osteocyte-specific (**D**) NRF2 binding sites. (**E**,**F**) Genome browser view of NRF2 binding in osteocytes and astrocytes at the *Ppard* locus (**E**) and the *Cd63* locus (**F**).

Beyond osteocytes, NRF2 has been implicated in the homeostasis of astrocytes [26] and endothelial cells [27]. To further elucidate the regulatory role of NRF2 in osteocytes, we compared NRF2 binding profiles between these cell types. Our analysis revealed both shared and cell-type-specific NRF2 binding sites (Figure 5A and Figure S2), suggesting shared and unique GRN under a cellular context. The results showed that shared NRF2 binding sites were involved in oxidant detoxification pathways (Figure 5B and Figure S2A). In contrast, cell-type-specific NRF2 binding sites indicated distinct roles tailored to each cell type, such as negative regulation of homotypic cell–cell adhesion in astrocytes or cell adhesion in endothelial cells (Figure 5C and Figure S2B) and regulation of carbohydrate metabolism and secretion in osteocytes (Figure 5D and Figure S2C). For instance, PPARδ, which is involved in carbohydrate metabolism and regulates bone mass [28,29], was under regulation of mechanical loading-induced NRF2 binding specifically in osteocytes (Figure 5E and Figure S3A). Additionally, CD63, which is a marker of the exosome and plays a pivotal role in secretion under mechanical loading [30,31], harbored an osteocyte-specific NFR2 binding (Figure 5F and Figure S3B). Thus, mechanical loading enhanced NRF2 genomic binding specifically in osteocytes and remodeled the transcriptional profile crucial for both antioxidative response and metabolic homeostasis.

## 4. Discussion

**Figure 6:**
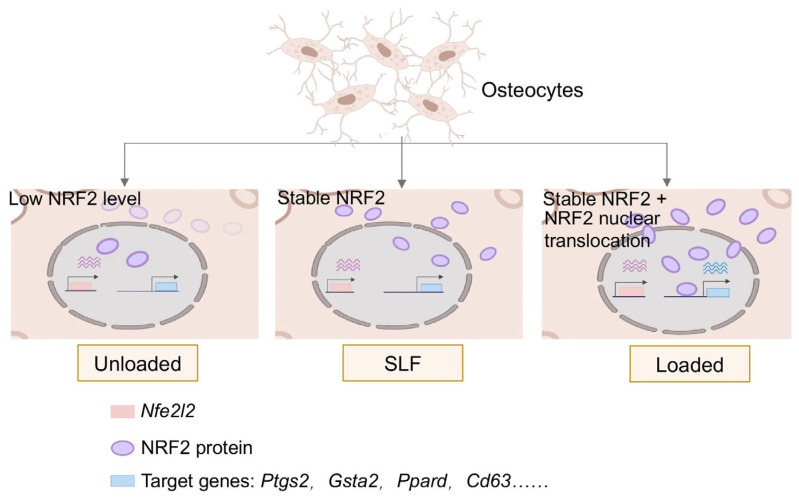
Mode of NRF2 regulation in osteocyte under mechanical loading. Left: Without mechanical loading, the NRF2 protein level was low in osteocytes. Middle: Under the treatment of SLF (NRF2 stabilizer), NRF2 protein was stabilized, and most remained in the cytoplasm of osteocytes. Right: Under mechanical loading, NRF2 protein was stabilized and translocated into the nucleus of osteocytes.

Our study revealed a novel mechanism by which mechanical loading enhanced NRF2 activation in osteocytes, facilitating both its stabilization and nuclear translocation. This mechanical activation of NRF2 drove unique epigenetic remodeling, promoting transcriptional programs that bolstered oxidative stress defense and maintained osteocyte homeostasis (Figure 6).

Previous studies have suggested that transcriptional levels of NRF2 mediate bone disease and exercise mitigation through DNA methylation in bone tissue [10]. However, in this study, we observed no transcriptional change in NRF2 in osteocytes. This was probably due to the differences between the study systems. Our work measured the effect of mechanical loading on NRF2 transcription in osteocytes only, while the previous work studied the femur tissue, which included osteocytes as well as the other cell types [10]. The transcription was measured as the overall level from osteocytes and the other cell types. It was previously reported that transcription of NRF2 would be increased in stem cells in the bone under mechanical loading [32]. Additionally, exercise changes the microenvironment in the bone through metabolism [33], which could affect the transcription in osteocytes indirectly. The effects of mechanical loading on NRF2 transcription in osteocytes in vivo need further investigation.

Instead of transcriptional changes, the NRF2 protein content in osteocytes significantly increased under mechanical loading, possibly due to enhanced translation or reduced protein degradation. However, only the NRF2 stabilizer sulforaphane (SLF) did not replicate the effects of mechanical loading. Unlike pharmacologic agents that stabilize NRF2, mechanical loading not only enhanced the protein level of NRF2 but also promoted its nucleus localization, a crucial step for its transcriptional regulatory function. Our findings showed that SLF could not mimic the effects of mechanical loading on gene expression, underscoring the complexity of mechanotransduction mechanisms. This highlights the need for multi-target therapeutic approaches in treating bone degenerative diseases. Currently, mechanical stimulation, such as whole-body vibration therapy, is applied clinically. The research in both animal models and humans demonstrated that vibration therapy could enhance bone strength and potentially treat bone-related diseases [34,35]. Furthermore, the studies suggest that combining vibration therapy with pharmacological interventions, such as vitamin D, an antioxidant and immunomodulator, can activate the Nrf2 signaling pathway and enhance therapeutic effects for conditions like osteoporosis [36,37]. Thus, integrating pharmacological treatments with mechanical stimulation could represent a more effective strategy for managing bone-related diseases, and this approach merits further clinical investigation.

Although our study revealed that nuclear translocation of NRF2 played a role mediating the effect of mechanical loading, the specific details for the nuclear translocation of NRF2 are not yet known. The previous studies have shown that focal adhesion and the permeability of nucleus pores can mediate the translocation of various transcription factors [38,39,40]. It has been demonstrated that mechanical force can stretch the nuclear pores complexes to increase permeability and reduce mechanical restriction of nuclear pores. This mechanism applies to force-triggered nuclear entry of various TFs, such as YAP [38,39]. Additionally, the nuclear localization of TFs such as YAP could also be a response to changes in F-actin polymerization and cell stretching under mechanical loading [40]. It is plausible that all these mechanisms could be involved in the nuclear translocation of NRF2. However, further studies are required to explore the exact mechanisms that facilitate NRF2’s nucleus translocation in response to mechanical loading.

Our study revealed the essential role of NRF2 in oxidative stress for osteocytes under mechanical loading. Previous studies have shown that NRF2 can also protect other mechanosensitive cells such as astrocytes and endothelial cells from oxidative stress [41,42]. By comparing the binding profiles of NRF2 in these cell types, we found that the regulation of NRF2 on oxidant detoxification pathways was shared among the cell types. Although the overall GRN mediated by the binding of NRF2 was specific under the cellular context, the regulation to prevent oxidative stress was shared. The results suggest regulation of oxidative stress response by NRF2 would be an essential and generalized function under mechanical loading in cell types.

Furthermore, there are some limitations of this study. Although our sequencing data showed good replications, a larger sample size would further enhance the robustness of our findings. In this study, we only investigated the short-term effects of mechanical loading for 12 h, while the long-term effects on osteocytes are still unclear. Previous research on periodontal ligament stem cells has shown that the activity of NRF2 under mechanical loading is persistent from 12 h to 36 h [43]. Additionally, endurance exercise through long-term and repetitive mechanical loading has been demonstrated to maintain bone homeostasis [44]. Thus, it is very possible that the effect of short-term mechanical loading can persist in long-term treatment. However, both the short-term and long-term effects of mechanical loading need to be examined in vivo by either animal models or human specimens to confirm the role of mechanical loading in osteocytes.

In conclusion, our study investigated the impact of mechanical loading on transcriptional and epigenetic remodeling in osteocytes. These results highlight the pivotal role of NRF2 in the mechano-responsiveness of osteocytes. Notably, the mechanical driving of NRF2-mediated oxidative stress defense reprogramming cannot be replicate by pharmacologic agents that stabilize NRF2. This underscores the irreplaceable role of mechanical stimuli, such as exercise, in promoting osteocyte oxidative homeostasis.

## Data Availability

RNA-seq data have been deposited at GEO (GSE273537) with the security token “qnoniqykxpijnex”. ChIP-seq data have been deposited at GEO (GSE273535) with the security token “srczywssrzahlwl”.

## Supplementary Materials

The following supporting information can be downloaded at https://www.mdpi.com/article/10.3390/antiox14030346/s1: Table S1: qPCR primers used in this study; Figure S1: The expression level of *Ptgs2* and *Gsta2*; Figure S2: NRF2 exerts cell-type specific role between human aortic endothelial cells and osteocytes; Figure S3: The expression level of *Ppard* and *Cd63*.
