# Supplementary material for: Mechanical Loading Induces NRF2 Nuclear Translocation to Epigenetically Remodel Oxidative Stress Defense in Osteocytes
Source: Antioxidants (Basel). 2025 Mar 15;14(3):346. doi: 10.3390/antiox14030346 (PMC11939503; doi:10.3390/antiox14030346)

Supplemental Table S1: qPCR primers used in this study.

Primer	Sequence
*Gsta2*_qpcr_F	AGGACTCTCACTAGACCGTGA
*Gsta2*_qpcr_R	CCCGGGCATTGAAGTAGTGA
*Slc7a11*_qpcr_F	GAGTGTCAGCTGGAGTGCCC
*Slc7a11*_qpcr_R	AAAGTTGAGGTAAAACCAGCCAGC
*Ptgs2*_qpcr_F	TGAGTACCGCAAACGCTTCT
*Ptgs2*_qpcr_R	CAGCCATTTCCTTCTCTCCTGT

Supplemental Figure S1: The expression level of Ptgs2 and *Gsta2* . (A) *Ptgs2* gene expression assessed by RNA-seq, normalized by TPM. (B) *Gsta2* gene expression assessed by RNA-seq, normalized by TPM. Data are mean ± SEM;

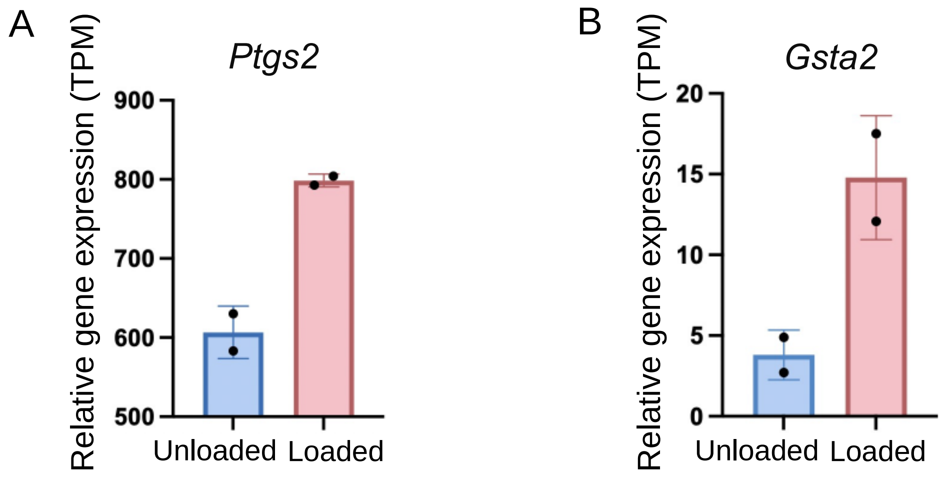

Supplemental Figure S2: NRF2 exerts cell-type specific role between human aortic endothelial cells and osteocytes. Gene ontology and pathway enrichment of shared (A) HAEC-specific (B) and osteocytes-specific (C) NRF2 binding sites.

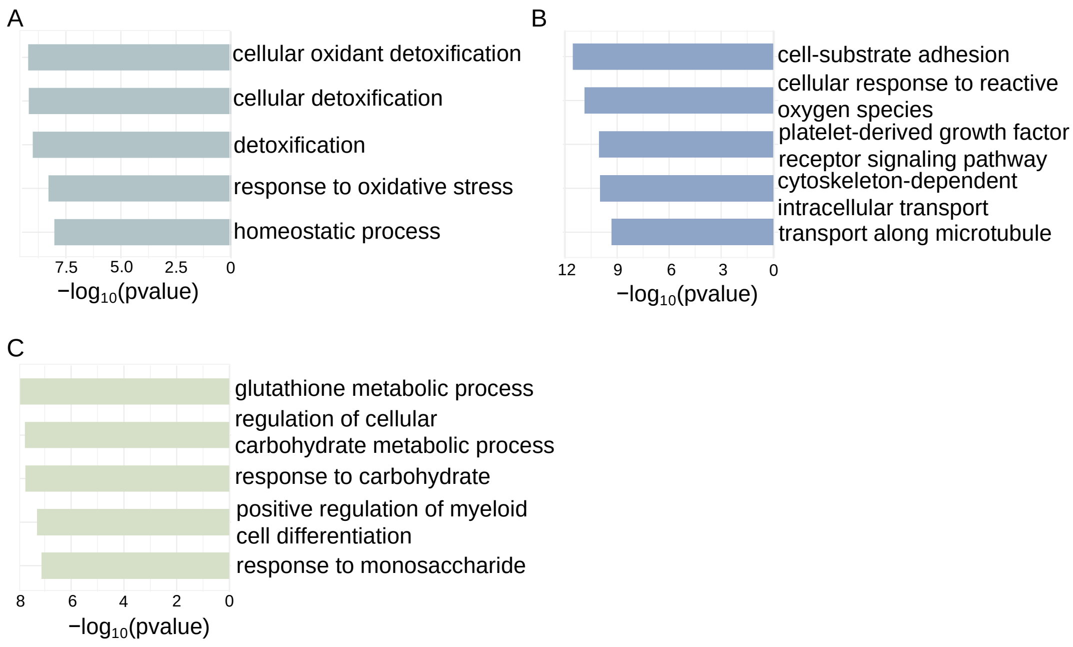

Supplemental Figure S3: The expression level of *Ppard* and *Cd63*. (A) *Ppard* gene expression assessed by RNA-seq, normalized by TPM. (B) *Cd63* gene expression assessed by RNA-seq, normalized by TPM. Data are mean ± SEM;

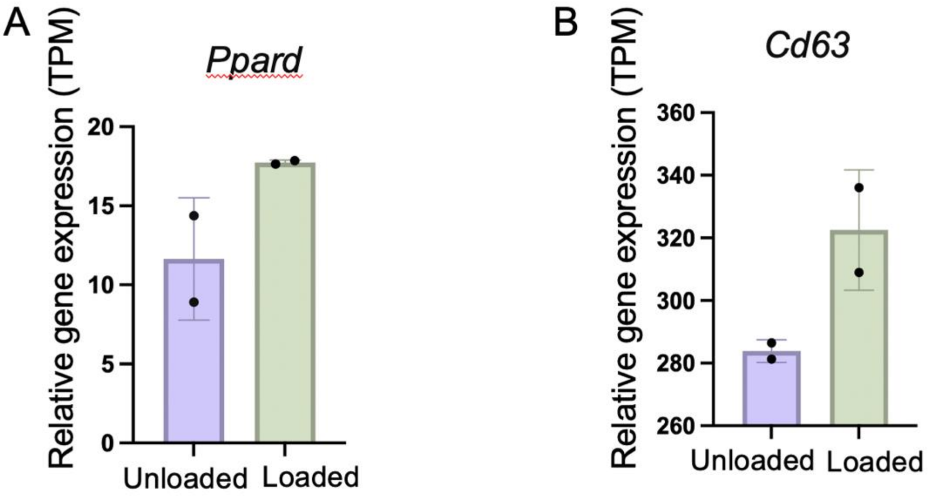

Supplement: Supplementary file 1 [file antioxidants-14-00346-s001.zip › antioxidants-3482555-supplementary.pdf]
